# IFIT3 activation significantly contributes to HIV-1-associated neurodegenerative disorder-mediated neuroinflammation

**DOI:** 10.3389/fimmu.2025.1532318

**Published:** 2025-08-27

**Authors:** Ranjit Kumar Das, Nirakar Sahoo, Deepa Roy, Chun Xu, Jose Galarza, Maria Camila Mejia Garza, Hansapani Rodrigo, Asim K. Duttaroy, Upal Roy

**Affiliations:** ^1^ Department of Health and Biomedical Sciences, University of Texas Rio Grande Valley, Brownsville, TX, United States; ^2^ School of Integrative Biological and Chemical Sciences, University of Texas Rio Grande Valley, Edinburg, TX, United States; ^3^ School of Mathematical and Statistical Sciences, University of Texas Rio Grande Valley, Edinburg, TX, United States; ^4^ Department of Nutrition, Institute of Basic Medical Sciences, Faculty of Medicine, University of Oslo, Oslo, Norway

**Keywords:** IFIT3, STAT1, cART, HIV, Tat protein, HAND, SH-SY5Y cells

## Abstract

**Introduction:**

The advent of effective combination antiretroviral therapy (cART) has significantly improved HIV-1 treatment, saving millions of lives. However, HAND remains a concern, particularly among aging individuals with HIV-1. The mechanisms underlying HAND are not well understood.

**Methods:**

This study investigated the role of interferon-induced protein with tetratricopeptide repeats 3 (IFIT3) and its upstream regulator, signal transducer, and activator of transcription 1 (STAT1), in HAND pathology. Using the SH-SY5Y neuroblastoma cell line and HIV-infected humanized mice, we examined the effects of the cART drugs, HIV Tat protein, and HIV-1 virus on STAT1 and IFIT3 expression.

**Results:**

The results showed that HIV-1 exposure significantly upregulated STAT1 and IFIT3, contributing to neuroinflammation.

**Discussion:**

This study identified IFIT3 as a critical molecular marker for HAND, suggesting its potential as a therapeutic target and offering new insights into disease pathology and treatment strategies.

## Introduction

Human immunodeficiency virus 1 (HIV) weakens the immune system of humans and thus has become a contributor to morbidity and the sixth leading cause of mortality worldwide (1). The World Health Organization (WHO) estimated that 39 million people were infected with HIV worldwide in 2022 (2). However, cART has significantly changed HIV pathology, making long-term management possible. cART involves multiple medications to inhibit the replication of the virus in the body (3), improving patients’ quality of life while living with the infection. However, in recent findings, some limitations of cART were identified, including drug toxicity, drug interactions, and the development of drug resistance, which have some adverse effects on cognitive function, such as mild cognitive impairment (MCI) and dementia (4, 5). Although cART effectively manages to control HIV infection, the occurrence of HAND, also referred to as HIV-associated Brain injury (HABI), remains prevalent, and the underlying mechanisms for this relationship remain poorly understood (6). Multiple factors are involved in the development of HAND, such as damage to neurons and impaired neurogenesis, which are mediated via several host and viral factors (3, 7). cART-mediated neuronal dysfunction is also thought to be responsible for the development of HAND in People with HIV (PWH) despite its ability to control viruses and prolong life (8). In addition, the viral protein Nef and certain other antiretrovirals cause autophagy, leading to neuronal dysfunction, cognitive impairment, and HAND (9). Therefore, the presence of HAND despite cART may be due to various combined factors causing dysregulated cellular processes in the central nervous system (CNS) (10). The JAK-STAT1 pathway is linked to HAND by activating interferon-gamma (IFN-gamma) and mediating neurotoxicity caused by the gp120 and Tat proteins (11). Activating the JAK-STAT1 pathway upon viral infection stimulates IFIT3. It activates mitochondrial antiviral-signaling protein (MAVS), forming signaling complexes that initiate a cascade of events, ultimately activating transcription factors such as NF-κB and the interferon regulatory factor (12). These transcription factors are translocated to the nucleus and induce the expression of genes encoding various proinflammatory cytokines, including type I interferons (IFNs), interleukins (ILs), and tumor necrosis factor (TNF), leading to increased production of proinflammatory cytokines that can initiate an inflammatory response (13, 14). As a result, immune cells in the CNS, microglia, and astrocytes can become activated and release cytokines and chemokines (15). This initiates a chain reaction in which immune molecules signal to each other, creating a loop that amplifies and sustains inflammatory responses. As a result, these inflammatory processes damage nerve cells and disrupt their communication ability, leading to various neurodegenerative and neuropsychiatric disorders (16). Persistent neuroinflammation despite cART is a significant comorbid condition for PWH (17). Therefore, identifying new biomarkers at the early stages of HAND development is imperative. Based on our previous observations, the present study aims to analyze further how cART drugs and HIV-1 impact the STAT1 and IFIT3 genes in HAND- and HIV-induced neuroinflammation. In the present study, we established a link between the expression levels of these genes in *in vitro* neuronal cells and in HIV-infected humanized mouse models. The potential outcome of this investigation is establishing a substantial connection between the expression of the STAT1 and IFIT3 genes and the severity of HAND. Therefore, these findings may pave the way for novel therapeutic approaches targeting these specific molecular pathways.

## Results

### Effects of antiretroviral drugs on the gene expression and viability of SH-SY5Y cells

**Figure 1:**
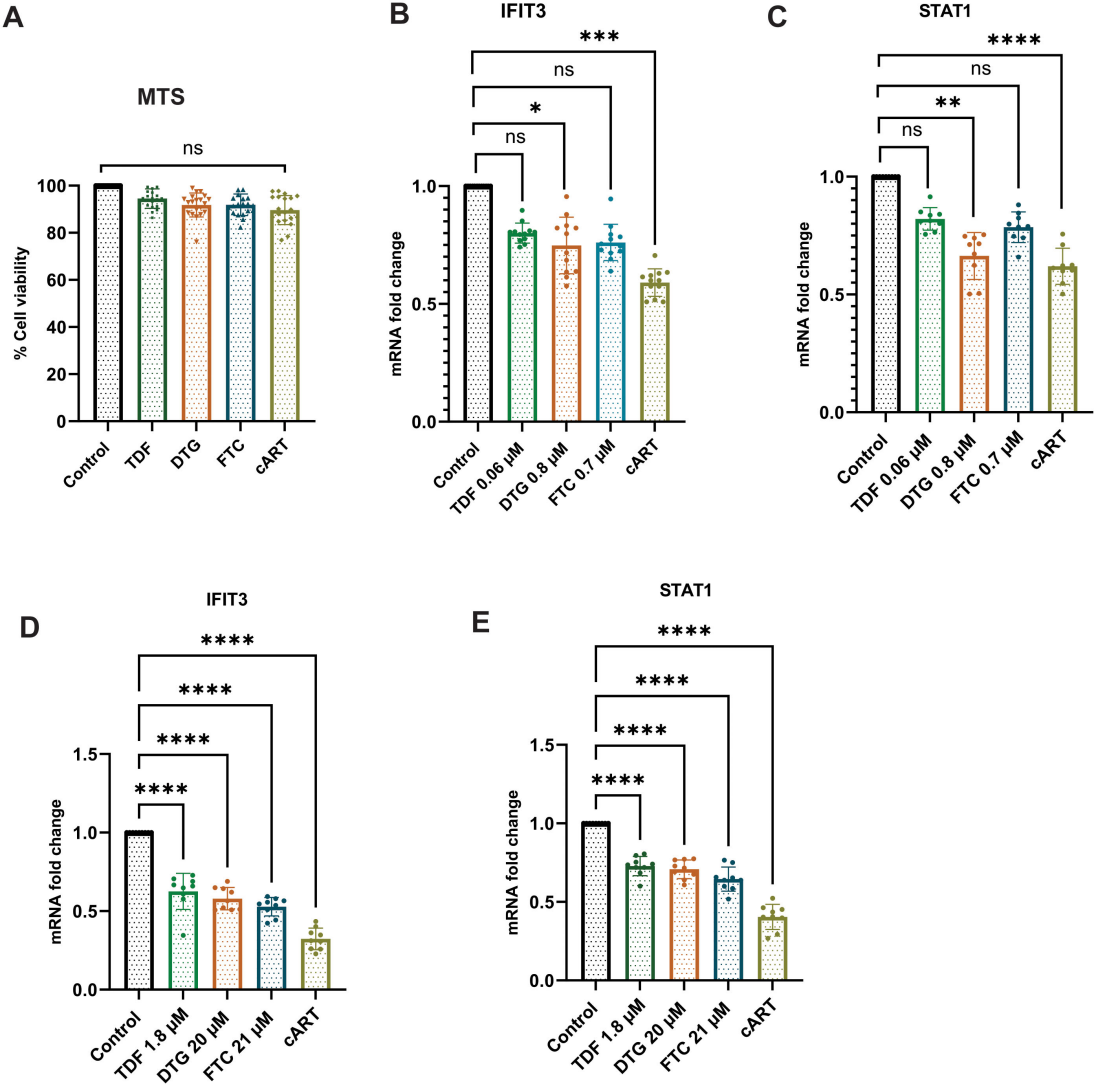
Effects of antiretroviral drugs on IFIT3 and STAT1 gene expression and cell viability in differentiated SH-SY5Y cells. Effects of individual and combined antiretroviral drugs on cell viability and gene expression in differentiated SH-SY5Y cells. **(A)** Cell viability assessed by MTS assay after 24-hour treatment with tenofovir disoproxil fumarate (TDF, 1.8 μM), dolutegravir (DTG, 20 μM), emtricitabine (FTC, 21 μM), or their combination (cART). **(B, C)** Relative mRNA expression levels of IFIT3 and STAT1, respectively, in cells treated with lower drug concentrations (TDF at 0.06 μM, DTG at 0.8 μM, FTC at 0.7 μM). **(D, E)** Relative mRNA expression levels of IFIT3 and STAT1 in cells treated with higher drug concentrations (TDF at 1.8 μM, DTG at 20 μM, FTC at 21 μM). Gene expression was quantified by RT-qPCR, with fold changes calculated relative to untreated control samples. MTS assay results represent the percentage of viable cells compared to control. Independent experiments included n = 9 replicates for RT-qPCR and n = 18 replicates for MTS assay. Statistical significance was determined by one-way ANOVA followed by Dunnett’s multiple comparisons test. *P < 0.0177; **P < 0.0015; ***P < 0.0011; ****P < 0.0001; ns, not significant.

This study investigated the effects of acute exposure to clinically relevant concentrations of antiretroviral drugs, both separately and in combination, on IFIT3 and STAT1 gene expression. The main goal was to create an *in vitro* model for HIV infection and antiretroviral treatment to determine which concentrations of the drugs effectively suppressed IFIT3 and STAT1 gene expression. We assessed the viability of differentiated SH-SY5Y cells exposed to clinically relevant concentrations of antiretroviral drugs using the MTS assay. Concentrations tested included Tenofovir Disoproxil Fumarate (TDF) at 1.8 μM, Dolutegravir (DTG) at 20 μM, and Emtricitabine (FTC) at 21 μM. Results showed no significant cytotoxic effects at these concentrations, indicating that TDF, DTG, and FTC do not significantly affect the viability of neuronal cells. These findings supported their selection for further experimental analysis (**Figure 1A**). Two different concentrations based on their clinical evidence of antiretroviral drugs were used for the *in vitro* study (18). To achieve this goal, the current study used drugs at their lowest therapeutic concentrations of TDF (0.06 μM), DTG (0.8 μM), and FTC (0.7 μM), and another set of highest therapeutic concentrations of TDF (1.8 μM), DTG (20 μM), and FTC (21 μM). We initially selected the lowest concentrations of the antiretroviral drugs mentioned above and cART drugs (a combination of the three drugs mentioned above at that concentration) to observe their effects on differentiated SH-SY5Y cells. The results indicated significant downregulation of the expression of the IFIT3 gene in the presence of DTG and cART within 24 hours of exposure (**Figure 1B**). However, the expression of the IFIT3 and STAT1 genes did not significantly differ between the cells exposed to TDF or FTC and the control cells. The effects of these antiretroviral drugs at the same concentrations on STAT1 gene expression were also investigated. Our data revealed that DTG (0.06 μM) significantly altered gene expression, with an approximately 0.8-fold change compared to the control (p = 0.0077). Similarly, acute exposure to the cART drugs significantly decreased STAT1 gene expression, with a 0.6-fold change compared to that of the control (p <0.0001) (**Figure 1C**). Overall, cART has a substantially greater effect on the downregulation of IFIT3 and STAT1 gene expression than individual drugs. Consequently, we investigated the effects of higher concentrations of these antiretroviral drugs, specifically TDF (1.8 μM), DTG (20 μM), FTC (21 μM), and cART drugs, on differentiated SH-SY5Y cells. Acute exposure to all antiretroviral drugs for 24 hours resulted in significant downregulation of IFIT3 genes with individual exposure or exposure to cART. There was a significant decrease in gene expression compared to the control group (p <0.0001) (**Figure 1D**). Similarly, the expression of the STAT1 gene was also significantly decreased compared to that of the control with the same concentrations of cART treatment (**Figure 1E**). Based on these findings, further experiments were designed with optimized concentrations of TDF (1.8 μM), DTG (20 μM), and FTC (21 μM).

### HIV and Tat upregulate IFIT3 and STAT1 gene and protein expression in SH-SY5Y cells

**Figure 2:**
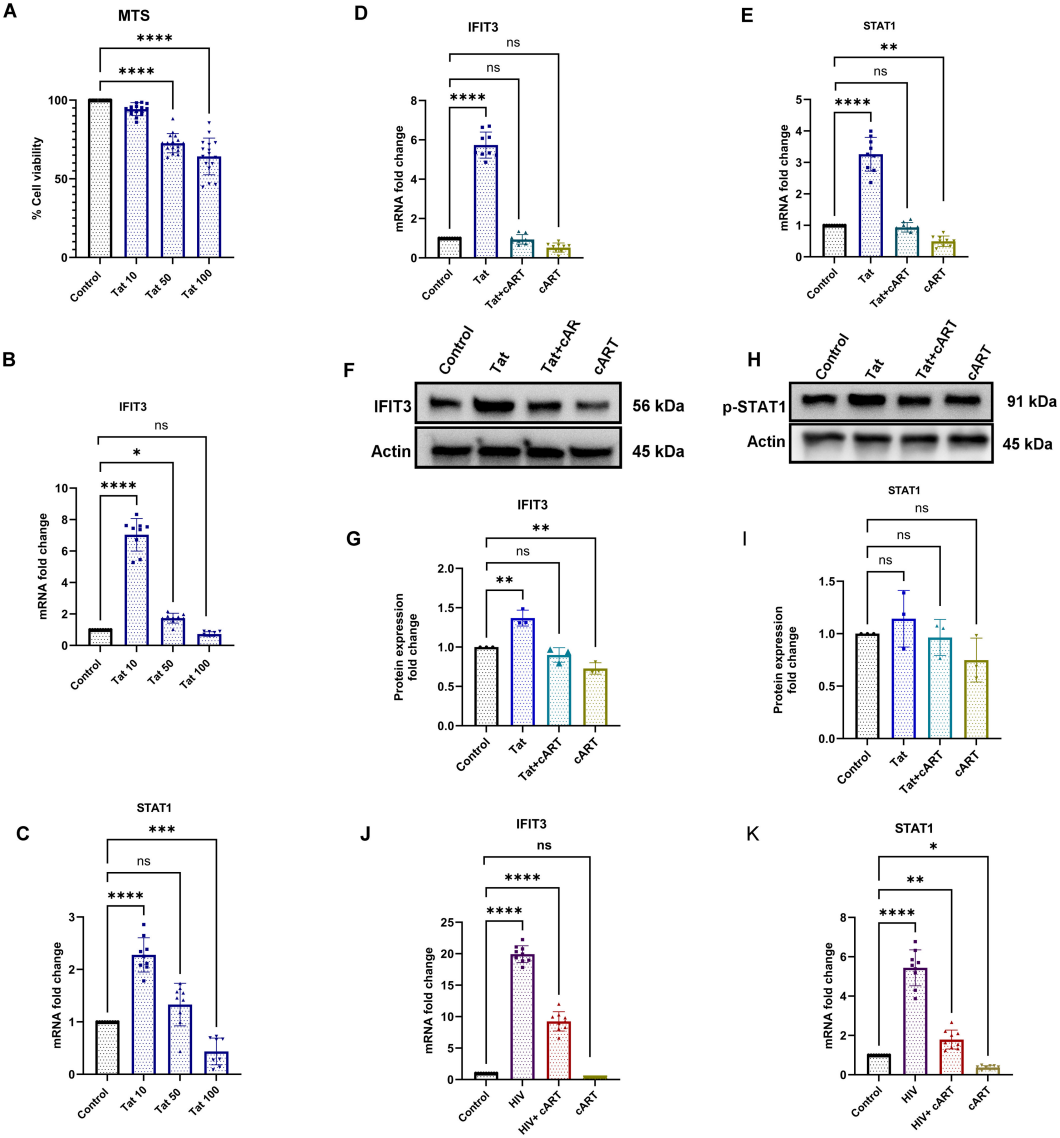
Effects of Tat and cART on IFIT3 and STAT1 gene expression and cell viability in differentiated SH-SY5Y cells. Effects of HIV-1 Tat protein and cART on SH-SY5Y cell viability, gene expression, and protein levels of IFIT3 and STAT1. **(A)** Cell viability of differentiated SH-SY5Y cells following treatment with 10, 50, and 100 ng/mL Tat protein for 24 hours, assessed by MTS assay. **(B, C)** Relative mRNA expression levels of IFIT3 and STAT1 following Tat treatment, evaluated by RT-qPCR. **(D, E)** IFIT3 and STAT1 gene expression levels after treatment with cART alone or combined Tat+cART treatment. **(F, H)** Representative Western blot images showing IFIT3 and STAT1 protein levels under different treatment conditions. **(G, I)** Densitometric analysis of IFIT3 and STAT1 protein expression normalized to β-actin. **(J, K)** Gene expression analysis of IFIT3 and STAT1 in SH-SY5Y cells treated with HIV, HIV+cART, or cART alone. Fold changes were calculated based on Ct values relative to untreated controls. Western blot quantification was performed using ImageJ, normalizing protein band intensities to β-actin. Independent experiments were performed for RT-qPCR (n = 9), MTS assay (n = 18), and Western blot analysis (n = 3). Statistical significance was determined using one-way ANOVA followed by Dunnett’s multiple comparisons test. *P < 0.05; **P < 0.0082; ***P < 0.001; ****P < 0.0001; ns, not significant.

SH-SY5Y cells were exposed to different concentrations of the HIV-Tat protein to observe the effects of the HIV protein on cell viability. The HIV-Tat protein stock was diluted with the culture medium as a working solution to ensure proper dosing. The Tat protein used its transduction domain to cross cell membranes. Once inside the cells, it interacts with transcription factors and modulates the activity of genes like IFIT3 and STAT1 which are vital for responding to viral infections. The 24-hour exposure to the Tat protein indicated a dose-dependent cytotoxic effect of the Tat protein on differentiated SH-SY5Y cells. Compared to the control, the lowest concentration (10 ng/ml) of Tat protein did not significantly affect the viability of differentiated SH-SY5Y cells. However, higher concentrations (>10 ng/ml) of Tat protein led to significant cellular toxicity. Following treatment with 50 ng/ml Tat protein, a significant reduction in cell viability (p <0.0001) was observed after 24 h of exposure. The trend of increasing toxicity was even more evident after exposure to the highest concentration of Tat protein (100 ng/ml) (**Figure 2A**).

To determine the most effective and nontoxic concentration of Tat protein in neuronal cells, we used three concentrations. Compared to the control, 10 ng/ml HIV-Tat significantly increased IFIT3 gene expression, which was approximately seven times greater (p < 0.0001). At 50 ng/ml, IFIT3’s expression increased nearly 2-fold (p = 0.0206), whereas at 100 ng/ml, IFIT3’s expression did not significantly change (**Figure 2B**). Therefore, 10 ng/ml was used for subsequent experiments.

In addition to IFIT3, we investigated the effects of Tat concentration on STAT1 gene expression. Using the same Tat concentrations, we found that 10 ng/ml significantly increased STAT1 expression, with levels twice as high as those of the control (p < 0.0001). Higher concentrations, such as 100 ng/ml, resulted in the downregulation of STAT1 expression (p < 0.0007) (**Figure 2C**). Thus, a Tat concentration of 10 ng/ml was chosen for the remainder of the study, as it significantly changed both IFIT3 and STAT1 gene expression.

The effects of the HIV Tat protein on IFIT3 and STAT1 gene expression were investigated in differentiated SH-SY5Y cells. Compared to the control, exposure to 10 ng/ml HIV-Tat resulted in a 5.7-fold increase in IFIT3 gene expression (p < 0.0001). However, the presence of cART drugs significantly suppressed IFIT3 gene expression both independently and in combination with the Tat protein (**Figure 2D**). Similarly, STAT1 gene expression was upregulated 3.2-fold in the presence of the Tat protein alone (p < 0.0001). When Tat was combined with cART or when cART was used alone, STAT1 gene expression was significantly downregulated (p = 0.0024) (**Figure 2E**). Western blot analysis was performed to evaluate the protein levels of IFIT3 and STAT1 following 24-hour treatments with Tat, Tat + cART, or cART alone. Tat protein exposure resulted in a significant increase in IFIT3 protein expression (p = 0.0010), whereas combined Tat and cART treatment suppressed IFIT3 protein expression. When treated with only cART, the IFIT3 protein level was significantly lower (p = 0.0068) (**Figures 2F, G**). In contrast, STAT1 protein expression was not significantly altered by any of the treatments. Western blot densitometry analysis confirmed that there was no significant expression of Tat protein levels compared to the control (**Figures 2H, I**). To assess the impact of HIV-1 on cytokines (IL-10), differentiated SH-SY5Y cells were exposed to HIV-1, HIV-1 with cART, or cART alone for 24 hours. Compared to the control treatment, HIV-1 exposure led to significant upregulation of IFIT3 gene expression, with a 20-fold increase (p < 0.0001). The combination of HIV-1 and cART significantly inhibited IFIT3 gene expression (p < 0.0001) but still remained significantly higher than control, whereas cART alone downregulated IFIT3 expression (**Figure 2J**). STAT1 gene expression was similarly affected, with HIV-1 alone causing a 5.7-fold increase (p < 0.0001). Compared to the control, HIV-1 + cART resulted in a 2-fold increase, which was less noticeable than that of HIV-1 alone (p = 0.0077). cART alone downregulated STAT1 expression (p = 0.0377) (**Figure 2K**).

### Tat induces the expression of the intracellular proteins IFIT3 and STAT1

**Figure 3:**
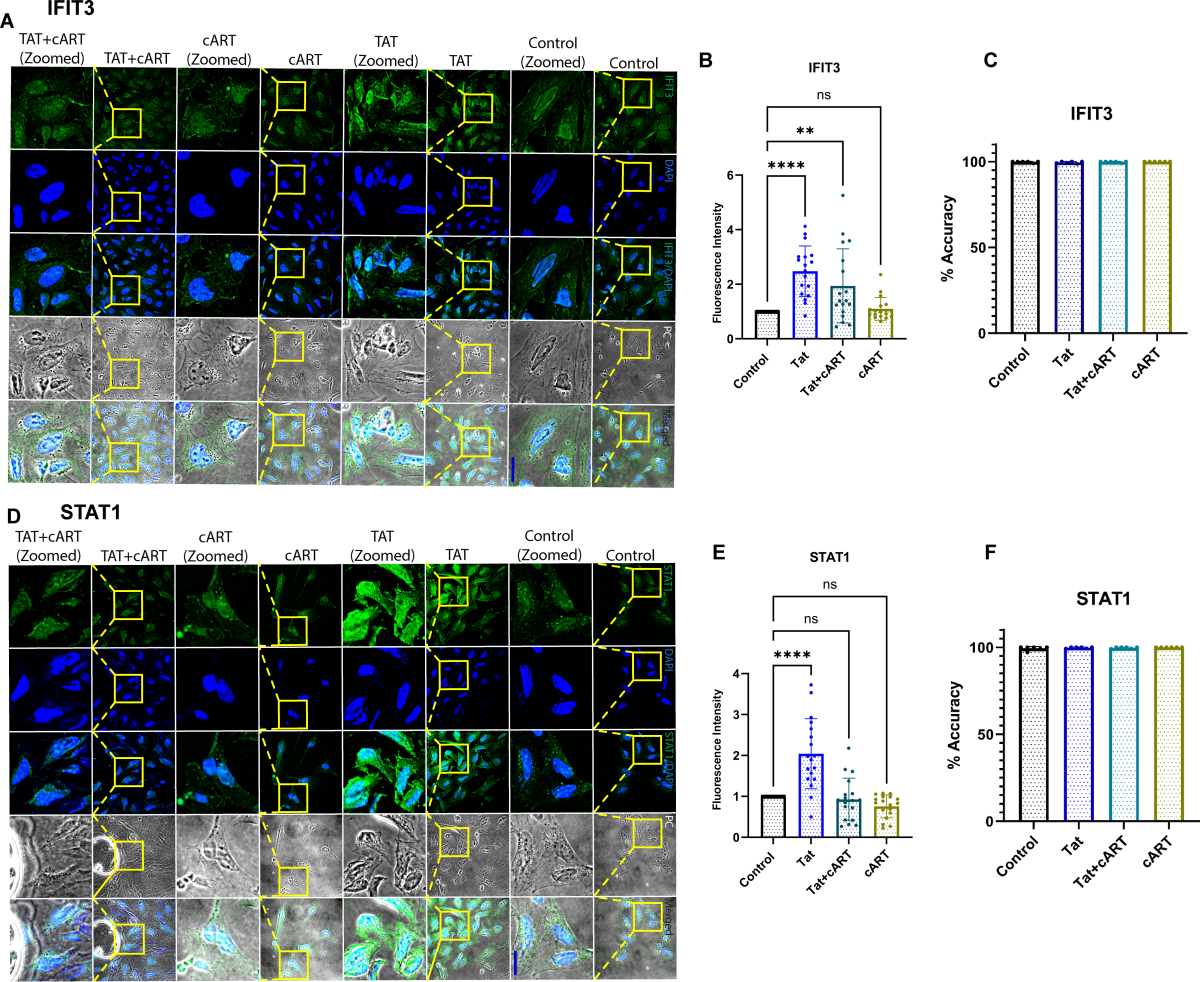
Immunocytochemical analysis of IFIT3 and STAT1 protein expression in differentiated SH-SY5Y cells. Immunocytochemical analysis of IFIT3 and STAT1 expression in differentiated SH-SY5Y cells following HIV Tat and cART treatments. **(A, D)** Representative confocal microscopy images showing IFIT3 and STAT1 protein expression in cells treated with HIV Tat protein, combination cART, or Tat+cART for 24 hours. IFIT3 and STAT1 were detected using Alexa Fluor 488-conjugated antibodies (green), and nuclei were counterstained with DAPI (blue). Phase contrast images detail cellular morphology, and composite images combine all fluorescence channels. **(B, E)** Quantitative analysis of IFIT3 and STAT1 expression under different treatment conditions. Data were collected from three independent experiments, with 5–6 images per experiment and 5–10 cells analyzed per image. **(C, F)** Machine learning analysis evaluating classification accuracy based on IFIT3 and STAT1 expression patterns. Statistical significance was assessed using one-way ANOVA followed by Dunnett’s multiple comparisons test. *P < 0.05; **P < 0.0027; ****P < 0.0001; ns, not significant.

Following the gene expression study, the effects of the HIV tat protein on the IFIT3 and STAT1 protein expression in differentiated SH-SY5Y cells were observed. In this regard, immunocytochemical studies were performed to examine the protein expression of the IFIT3 and STAT1 proteins after exposure to Tat, Tat + cART, or cART alone. This experiment was carried out to understand the possible regulatory impacts of HIV-Tat and cART on the expression of the IFIT3 and STAT1 proteins. Following 24 hours of HIV-Tat treatment, we observed a significant increase in the immunocytochemical staining intensity for IFIT3 (p <0.0001) (**Figures 3A, B**) and STAT1 (p <0.0001) (**Figures 3D, E**), suggesting an increase in their relative abundance. These results suggest that Tat exposure alone increased the protein expression of IFIT3 and STAT1. However, protein expression significantly differed when the cells were treated with the Tat+cART. Interestingly, we detected a moderate reduction in IFIT3 protein expression. When the cells were exposed to cART alone, the expression of the IFIT3 protein was reduced (p=0.9733) (**Figures 3A, B**), and the expression of the STAT1 protein was similar to that of the control, indicating a potential inhibitory effect of cART on the relative abundance of these proteins (**Figures 3D, E**). Additionally, automated image analysis via AI demonstrated that the classification accuracy for the IFIT3 (**Figure 3C**) and STAT1 (**Figure 3F**) treatments was approximately 100% on average across 6 runs per protein expression. As mentioned before, the improvement in classification accuracy was due to the use of a balanced dataset for training. The classification values in the IFIT3 and STAT1 test runs are 100% for all the control groups. The results revealed the importance of the balanced dataset, which can be further improved as more data is obtained.

### HIV-1 and Tat proteins upregulate cytokine expression and ROS production in SH-SY5Y cells

**Figure 4:**
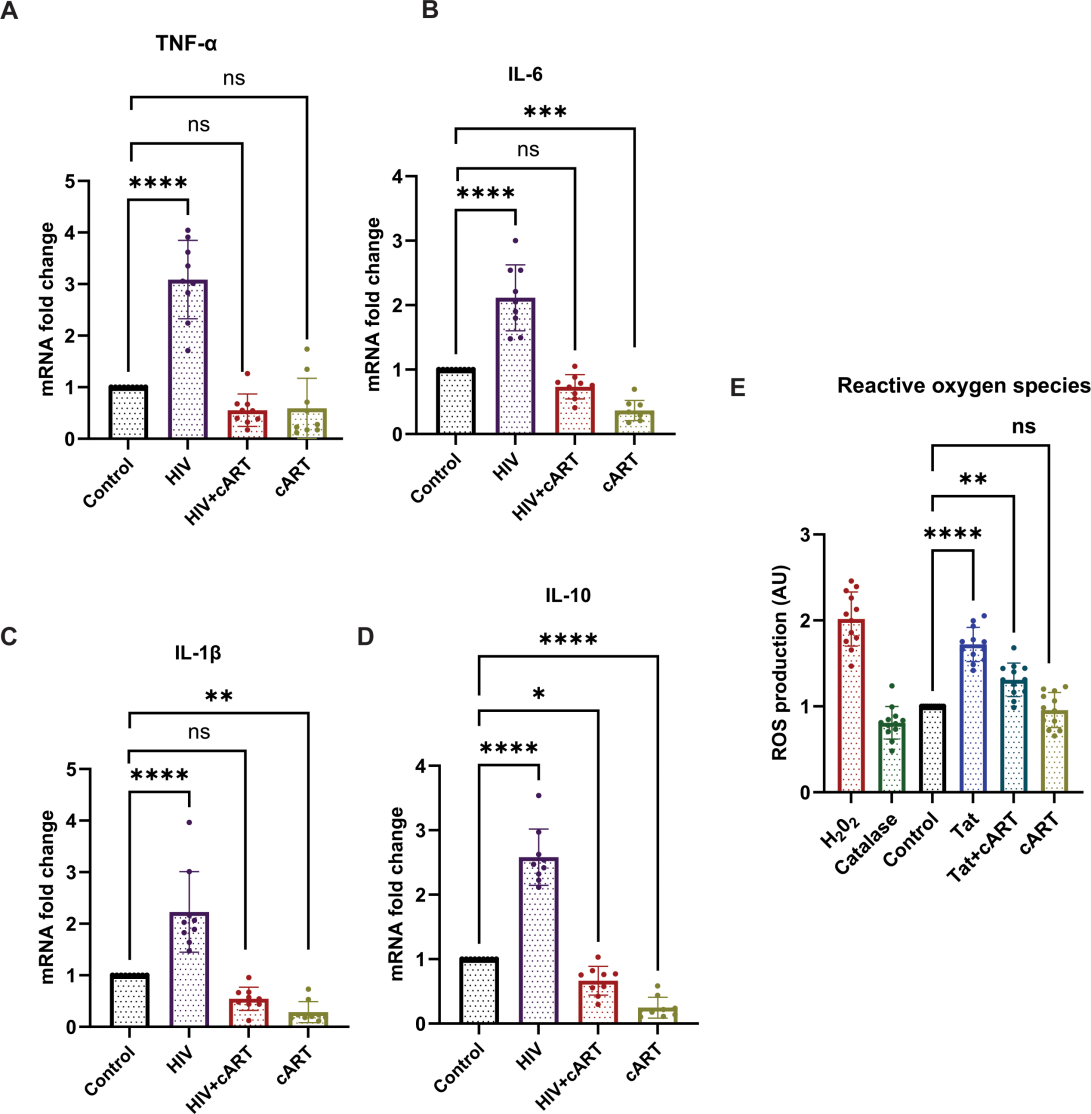
Effects of HIV on cytokine production and ROS induction in SH-SY5Y cells. HIV-1 exposure modulates cytokine expression and induces oxidative stress in differentiated SH-SY5Y cells. **(A–D)** mRNA expression levels of proinflammatory cytokines TNF-α, IL-1β, and IL-6, as well as the anti-inflammatory cytokine IL-10, were assessed by RT-qPCR following 24-hour treatment with HIV, HIV+cART, or cART alone, compared to untreated control cells. **(E)** Intracellular ROS levels were quantified using H_2_DCFDA fluorescence in control, Tat-treated, and Tat+cART-treated groups. Hydrogen peroxide (H_2_O_2_) served as a positive control for ROS induction, while catalase treatment served as a negative control. For cytokine gene expression analysis, three biological replicates (n=3) were used, and for ROS measurements, twelve replicates (n=12) were analyzed. Gene expression was normalized to GAPDH, and fold changes were calculated relative to control samples. Statistical significance was determined using one-way ANOVA followed by Dunnett’s multiple comparisons test. *P < 0.05; **P < 0.01; ***P < 0.001; ****P < 0.0001; ns, not significant.

To explore the effects of HIV exposure on cytokine expression in SH-SY5Y cells, SH-SY5Y cells were exposed to HIV, HIV+cART, or only cART. After 24 hours of exposure, cytokine expression was assessed. The results revealed a significant increase in the expression of proinflammatory cytokines (TNF-α, IL-6, and IL-1β) and anti-inflammatory cytokines (IL-10) in the HIV group compared to the control group (p <0.0001). On the other hand, in the HIV+cART group, the levels of IL-1β and IL-6 were significantly reduced (p < 0.0054 and p < 0.0014, respectively), demonstrating the efficacy of cART in mitigating the inflammatory effects induced by HIV. Compared to the control group, the cART group presented a significant change in cytokine expression, confirming that cART also independently affects cytokine levels (**Figures 4A-D**) Differentiated SH-SY5Y cells were further tested to observe the effect of HIV-Tat on Reactive oxygen species (ROS) production. The objective of this study was to determine changes in ROS generation after exposure to Tat, Tat + cART, and cART. Tat-treated cells exhibited the highest level of ROS production (19). The Relative Fluorescence Units (RFU), indicative of the level of ROS, was markedly greater in Tat-treated cells than in control cells (p <0.0001). On the other hand, the level of ROS production was lower in cells exposed to Tat + cART than in cells treated with Tat alone. However, it was still greater than that of the control (p <0.0006), indicating that cART treatment potentially reduces the ROS-generating effect of Tat in SH-SY5Y cells. The cART treatment alone did not significantly affect ROS production compared to the control (**Figure 4E**).

### HIV-1 infection upregulates IFIT3 expression in humanized mice

**Figure 5:**
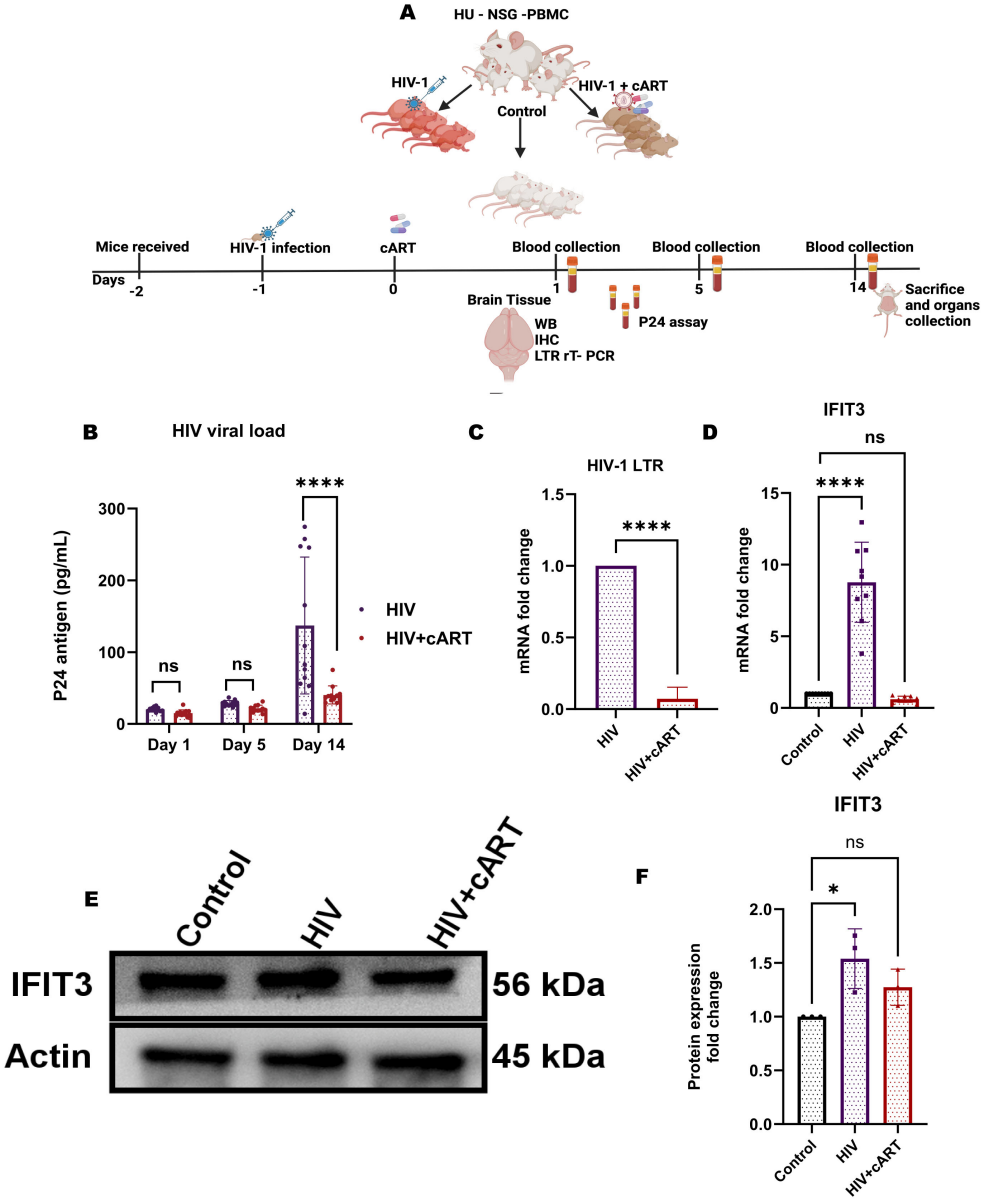
HIV-1 infection increases IFIT3 expression and viral burden in a humanized mouse model. **(A)** Experimental workflow: Humanized NSG-PBMC mice were engrafted with human PBMCs (Day -2), infected with HIV-1 (Day -1), and initiated on cART (Day 0). Three groups were established: control, HIV-infected, and HIV-infected + cART. Plasma samples were collected on Days 1, 5, and 14 for HIV-1 P24 quantification. On Day 14, brain tissues were harvested for RT-qPCR, Western blot, and immunohistochemistry. **(B)** Plasma P24 levels confirmed elevated systemic viral load in HIV-infected mice compared to controls. **(C)** Brain HIV-LTR expression was significantly higher in HIV-infected mice and partially reduced with cART. **(D)** IFIT3 gene expression was upregulated in brain tissues of HIV-infected mice and attenuated by cART treatment. **(E)** Western blot analysis showed increased IFIT3 protein expression with HIV infection and partial normalization following cART, with densitometric quantification **(F)** normalized to β - actin. Statistical significance was determined using one-way ANOVA with Dunnett’s multiple comparisons test for IFIT3 gene and protein expression and a t-test for HIV-LTR results. Significance is indicated as *p = 0.219, ****p < 0.0001, and ns, not significant.

**Figure 6:**
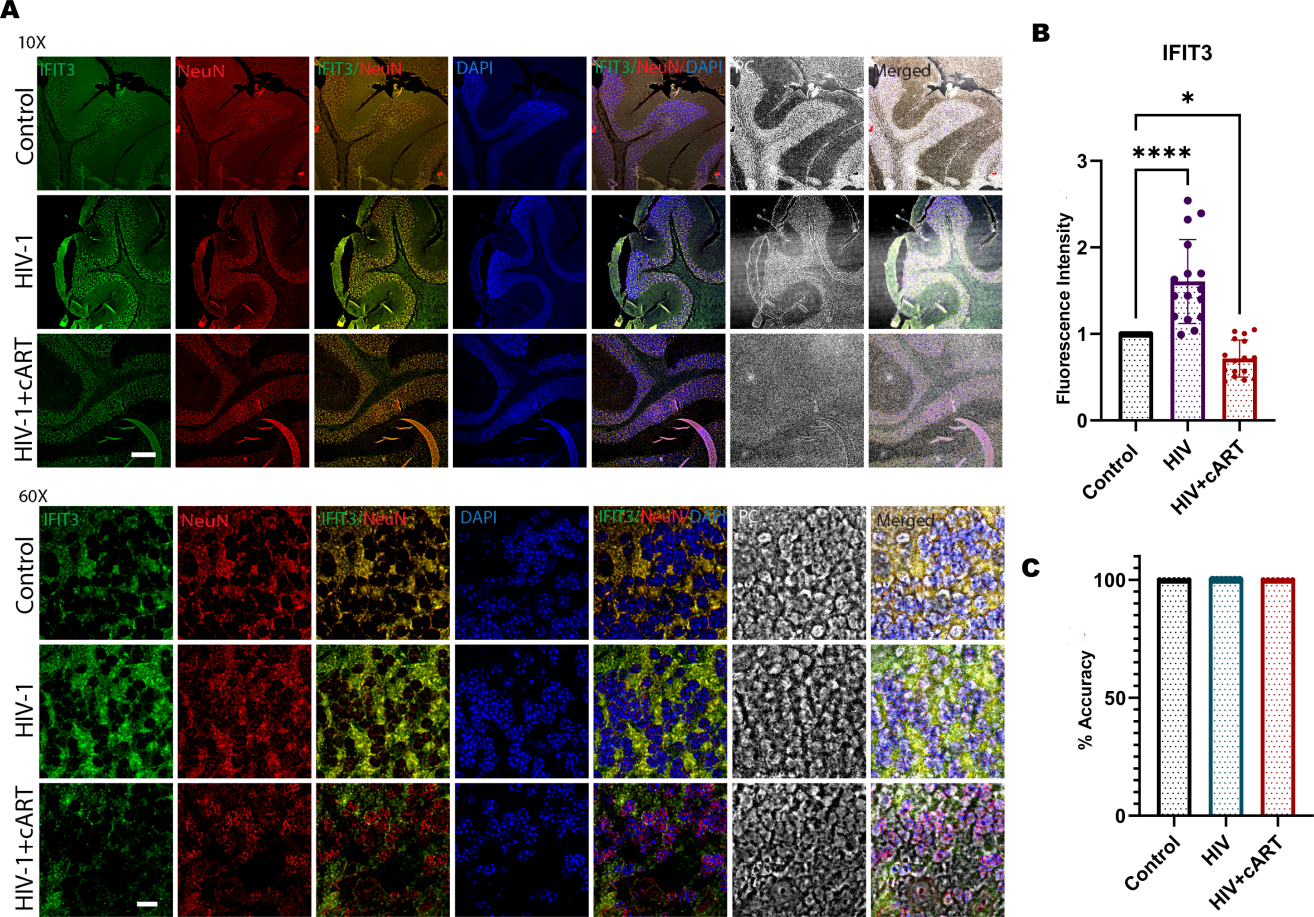
**(A)** Immunohistochemical analysis of cerebellum brain tissue from humanized mice subjected to HIV-1 and cART is shown in the remaining panels. Representative images illustrate IFIT3 expression (green fluorescence), neuronal cells labeled with the NeuN marker (red fluorescence), and nuclear staining with DAPI (blue fluorescence). Column 1 shows IFIT3 expression. Column 2, NeuN labeling; Column 3, DAPI staining; Column 4, a composite image of IFIT3, NeuN, and DAPI. Column 5 shows phase contrast (PC) imaging to detail the cellular morphology. Column 6 shows a composite overlay of IFIT3/NeuN/DAPI with phase contrast imaging to provide a holistic view of expression patterns within the cellular architecture. **(B)** shows the fluorescence intensity, and **(C)** shows the accuracy analysis via MATLAB. The study included brain tissue samples from six individual mice (n=6) in each treatment group.Differences in IFIT3 expression were statistically analyzed via one-way ANOVA with Dunnett’s multiple comparisons test. Significance levels are indicated as *p = 0.219, ****p < 0.0001 and ns, not significant.

To observe HIV infection and the effect of cART on this model, an HIV P24 antigen assay and Long Terminal Repeat (LTR) PCR were performed on plasma and brain samples from HIV-infected and cART-treated humanized mice, respectively. In this regard, the study has used hu-PBL mice considering its timely human immune system engraftment and HIV infection in this model (**Figure 5A**). Our previous study has shown that this model can establish HIV infection within 12 hours of HIV inoculation and the effect of the treatment can be observed within one week of administration (20, 21). Thus, this model was preferred and adopted for the study compared to other existing models for HIV infection where infection and disease pathologies take a longer time to establish HIV infection pathologies (20–23). The results of the p24 assay revealed a gradual increase in infection from day 1 to day 5, and more prominently, on day 14, the HIV group presented a significant increase in viral load, indicating successful infection in this model. In contrast, the HIV + cART group presented a substantial reduction in p24 levels (**Figure 5B**), demonstrating the ability of cART to suppress active HIV replication in these mice. Additionally, HIV-LTR PCR analyses revealed a significant reduction in the latent HIV LTR gene in the HIV+cART group compared to the HIV group (p-value <0.0001), as shown in **Figure 5C**. These findings suggest that cART not only inhibits active viral replication but also helps in reducing latent HIV in the brain, an indicator of effective cART. The present study also investigated the expression of the IFIT3 gene and protein in brain tissues from the humanized mouse model. The experiment was designed to clarify the expression of IFIT3 at the gene and protein levels in response to HIV infection and its modulation by cART. To evaluate the expression of the IFIT3 gene, we conducted gene expression analysis in three different experimental groups. Control, HIV-infected, and HIV + cART-treated mice. Our PCR analysis of IFIT3 gene expression revealed significant upregulation in the HIV-infected group compared to the control group, which was almost 7-fold greater than the control group (p< 0.0001), indicating a notable response to viral infection at the genetic level. In contrast, the group treated with HIV+ cART presented a reduction in IFIT3 gene expression, which was lower than that in the control group (**Figure 5D**). Following this promising finding at the gene expression level, Western blotting was used to assess IFIT3 protein levels, providing information on the posttranscriptional impact of HIV infection and cART treatment. Compared to control mice, the protein expression of IFIT3 was significantly greater in HIV-infected mice (p= 0.0219), underscoring the upregulation of gene expression and protein synthesis in the presence of HIV. In the HIV+cART-treated group, a significant decrease in the expression of the IFIT3 protein was observed indicating the efficacy of cART in negatively regulating IFIT3 protein expression levels. (**Figures 5E, F**). However, it was not significantly different from the control group suggesting that while cART effectively controls viral replication, its impact on IFIT3 protein expression was not significantly altered during the tested conditions. To gain a deeper understanding of IFIT3 protein expression in humanized mice, we conducted a series of immunohistochemical analyses. This approach allowed us to precisely quantify the IFIT3 protein within distinct neuronal populations in brain tissues derived from both the HIV-infected and control groups. This focus was guided by evidence suggesting that neurons are particularly susceptible to HIV, which can lead to HAND. IFIT3, a crucial component of the neuronal interferon response, plays a significant role in the antiviral defense mechanisms of neurons, making it a pertinent marker for studying the effects of HIV at the cellular level. By employing the neuronal marker NeuN, we specifically targeted our examination to neuronal cells, ensuring that our findings would accurately reflect the changes in IFIT3 expression due to HIV infection and its treatment with cART (p < 0.0001). Our results revealed a significant increase in IFIT3 expression in the HIV group compared to the control group, confirming the gene upregulation at both the gene and protein levels following HIV infection. In the HIV+cART group, IFIT3 expression was significantly downregulated (p= 0.0219), indicating the effectiveness of cART in reducing the expression of this gene in the context of HIV infection (**Figures 6A, B**). These findings suggest that cART may have a beneficial effect on mitigating the impact of HIV on neuronal cells by modulating the expression of IFIT3. This evaluation was further validated with a machine-learning approach. This can impact model accuracy, particularly for IFIT3 classification. Model accuracy for different treatments was quantified, with the control, HIV, and HIV+ cART treatments showing accuracies of 100% (**Figure 6C**). The confidence interval analysis, which was based on six runs with preliminary data, indicated the impact of data balance on classification accuracy. The results revealed that this improved dataset classification resulted in approximately 100% classification accuracy.

## Discussion

HAND remains a significant complication for the PWH, even in the era of cART. Despite effective viral suppression, many PWH continue to experience cognitive impairments due to chronic neuroinflammation and neuronal damage (24). Currently, there are no established gene-level biomarkers for detecting the early development of HAND, which limits timely intervention. This study characterized the role of the IFIT3 gene in HAND.

In our study, the *in vitro* concentrations of the antiretroviral drugs (TDF, DTG, and FTC) were carefully selected to reflect clinically relevant plasma therapeutic levels based on established pharmacokinetic data (25, 26). These concentrations were specifically chosen to mimic the systemic exposure achieved in patients receiving standard cART regimens, thus ensuring the translational relevance of our findings. Although we recognize that CNS drug concentrations may be lower than plasma levels due to limited blood–brain barrier permeability, particularly for TDF and FTC (27). Our primary aim was to model systemic drug effects rather than to replicate CNS pharmacokinetics. Moreover, DTG, one of the agents studied, is known to have moderate CNS penetration and was included to reflect drugs capable of impacting neuroinflammatory pathways within the brain. Therefore, the concentrations used in our experiments are consistent with clinically meaningful exposure and align with previous experimental models evaluating antiretroviral neurotoxicity and neuroprotection. Our findings show that clinically relevant lower concentrations of DTG (0.08 µM), FTC (0.7 µM), and TDF (0.06 µM) affect the expression of STAT1 and IFIT3 in SH-SY5Y cells, although they are not toxic at these concentrations. DTG, an integrase inhibitor, significantly altered gene expression, whereas FTC and TDF, nucleoside reverse transcriptase inhibitors, had no substantial effects on STAT1 or IFIT3 at these concentrations. This finding suggests that DTG might play a more prominent role in modulating interferon pathways, which are crucial for the antiviral response (28). The distinct effect of DTG at low concentrations likely arises from its role as an integrase inhibitor. This role may influence transcription processes and interact with endogenous retroviruses, differentiating it from other antiretrovirals (29) There is also evidence that indicated DTG can reduce inflammation by decreasing NFkB activation and secretion of IL-6 and IL-8 (30). Higher concentrations of these drugs could modulate the interferon pathway, reflecting their complex interactions with cellular processes and their potential to affect neuroinflammatory responses.

This study also examined the effects of the HIV Tat protein, which is pivotal in viral replication and transcription (31). Our results indicate that Tat significantly reduces cell viability at relatively high concentrations, which we link to increased levels of ROS and oxidative stress (32). At a concentration of 10 ng/ml, Tat increased the expression of STAT1 and IFIT3, suggesting a cellular defense response. Exposure to 100 ng/mL Tat resulted in the downregulation of STAT1 expression. We interpret this finding as an indication of cellular stress and transcriptional dysregulation rather than a direct physiological regulation of STAT1, consistent with previous studies showing that high concentrations of Tat can induce oxidative damage, mitochondrial dysfunction, and widespread transcriptional repression in neuronal cells (33, 34). In contrast, 10 ng/mL Tat significantly increased STAT1 and IFIT3 expression without inducing substantial cytotoxicity, supporting its use as a physiologically relevant concentration based on established experimental models. When Tat was combined with cART, the effects of Tat on these genes were mitigated, highlighting the role of cART in restoring disrupted cellular pathways (35). Our findings indicate that cART exerts an independent suppressive effect on IFIT3 and STAT1 expression in differentiated SH-SY5Y cells, as treatment with cART alone significantly reduced the expression of these genes compared to untreated controls. Furthermore, when cART was co-administered with Tat protein, the Tat-induced upregulation of IFIT3 and STAT1 was significantly attenuated but not at the level of control cells. These results suggest that cART can both independently suppress baseline gene expression and counteract Tat-mediated activation. Thus, cART exerts dual effects by mitigating both baseline and Tat-induced neuroinflammatory signaling pathways, which aligns with the role of the JAK/STAT pathway in immune response modulation (36). The introduction of cART reduced the expression of these genes, suggesting its efficacy in suppressing HIV replication and associated chronic inflammation (37). This finding was corroborated by protein expression analysis and immunocytochemistry, which revealed consistent modulation of IFIT3 and STAT1 by cART, strengthening its therapeutic objectives. However, elevated level of IFIT3 even in presence of HIV and cART indicated an increase risk of HIV associated neuropathology in the long-term. Our investigation of ROS production revealed that Tat exposure substantially increased ROS levels, indicating that oxidative stress is a significant factor in HIV-associated neurodegeneration (38). This finding aligns with the observation that oxidative stress exacerbates neuroinflammation, leading to further neuronal damage (39). When cART was co-administered with Tat, the ROS levels were lower than those in cells treated with only Tat, although they remained elevated compared to untreated cells. These findings indicate that cART can partially mitigate Tat-induced oxidative stress, but not at the level of untreated control which is a significant concern for PWH with cART. The use of a humanized mouse model infected with HIV provided further validation of our *in vitro* findings. We observed significant suppression of viral replication in the mouse brain with cART treatment, as evidenced by reduced levels of the HIV p24 protein. This suppression emphasizes the effectiveness of cART in managing the viral load, which is crucial for preventing the progression of HIV induced neuropathologies (40).

Additionally, the reduction in the viral genome in the mouse brain emphasizes the potential of cART in preventing the establishment of latent HIV reservoirs in the CNS (41). A pivotal aspect of our findings was the significant increase in IFIT3 expression in HIV-infected mice compared to that in controls, highlighting its role in the interferon-mediated immune response (42). IFIT3, an interferon-stimulated gene, enhances host antiviral defense mechanisms, reflecting a cellular attempt to curtail viral replication. The upregulation of IFIT3 in response to HIV-1 infection and subsequent reduction after cART treatment demonstrated the ability of cART to normalize immune function and reduce chronic immune activation, a key factor in HIV-associated neuroinflammation.

Our study uniquely characterized IFIT3 and STAT1 as central regulators of neuroinflammation in the context of HIV infection and cART treatment. Modulating these pathways with cART has the potential to restore immune homeostasis and reduce neuroinflammatory responses (43). This insight into the molecular mechanisms of HAND provides a foundation for developing targeted therapies that address viral suppression and neuroprotection. Furthermore, the observed cytokine production by IFIT3 in response to HIV infection and cART treatment provides additional information on the inflammatory pathways involved in HAND (44). The regulation of cytokines, such as TNF-alpha, IL-2, IL-6, and IL-10, by IFIT3 emphasizes its dual role in antiviral defense and its potential contribution to immune dysregulation when overexpressed (45, 46). Balancing effective antiviral responses with the risk of excessive inflammation highlights the complexity of managing neuroinflammation in PWH. The translational importance of our study lies in the identification of IFIT3 and STAT1 as potential therapeutic targets to reduce neuroinflammation in HIV-infected individuals receiving cART. Targeting these pathways could lead to novel interventions that specifically address the neurocognitive complications of HIV, improving patient outcomes. Future studies should explore the specific cell types and molecular mechanisms involved in these processes and the long-term effects of cART on neuronal health and function. This study characterized two key JAK/STAT pathway regulators that contribute significantly to chronic neuroinflammation and HAND pathology. Previous studies have indicated the upregulation of IFN-pathway genes during HIV infection in PWH (47–49). However, the understanding of the molecular mechanisms of neuroinflammation in these PWHs was largely unknown. Therefore, the present study is the first of its kind to study the effect of the IFIT3-induced interferon pathway in the presence of HIV and cART drugs.

This study highlighted key modulators, such as IFIT3 and STAT1, during HIV infection and cART, which could be therapeutic targets for the future treatment of HIV-associated neuroinflammation. This study also revealed that different types of brain cells, including neurons, are involved in chronic inflammation in the brain, which is quite common in cART-treated PWH, indicating the importance of this study. However, the study did not investigate the role of any specialized cells in the mouse model that contributed to this process, which needs further study. Throughout the study, it was also observed that there was some inconsistency in STAT1 expression level. This could be due to phosphorylation pattern and longer activation time as suggested by others (50). While IFIT3 expression was comprehensively evaluated in both *in vitro* and *in vivo* models, STAT1 expression was assessed only in *in vitro* experiments. Future studies should incorporate STAT1 analysis in animal tissues to further expand mechanistic insights into HAND-related neuroinflammation.

While we acknowledge that these *in vitro* and mouse model studies do not fully summarize the complex cART drug pharmacokinetics and neurocognitive impairment of humans, it provides foundational insights that are critical for understanding potential underlying mechanisms that could contribute to neurocognitive impairments. Thus, to our knowledge, this is the first evidence of deciphering the underlying molecular mechanism of chronic neuroinflammation in the HIV-infected brain in the presence of cART (23), which has significant translational importance.

## Materials and methods

### Reagents and antibodies

All reagents and antibodies were obtained from the following sources: SH-SY5Y neuroblastoma cells (catalog #CRL- 2266, ATCC). Eagle’s Minimum Essential Medium (EMEM) (Catalog # 30-2003, ATCC), Fetal Bovine Serum (FBS) (Catalog # SH30071.03IR25-40, Cytiva), Dulbecco’s modified Eagle’s medium (DMEM) with high glucose (Catalog # SH30022. FS, Cytiva) Dulbecco’s phosphate buffer saline (DPBS) 1× (catalog # SH30256, Cytiva), penicillin–streptomycin-glutamine (catalog # 10378016, Thermo Fisher), and all trans retinoic acid (RA) (catalog # 302-79-4, Millipore Sigma) were used. 2 - Beta mercaptoethanol (catalog: BP176-100), Dimethyl sulfoxide (DMSO) (catalog: 67-68-5, Fisher Scientific), TDF (catalog #202138-50-9), FTC (catalog #143491-57-0) from MilliporeSigma, MA, USA), DTG (catalog #1051375-16-6, Advanced ChemBlocks Inc., Hayward, CA, USA). HIV-1 Tat protein (catalog# 7002, Immunodx Woburn, MA, USA), RNase AWAY™ Surface Decontaminant (catalog # 7002), DEPC-treated water (catalog #AM9939), RNeasy Mini Kit (Cat: 74104, Qiagen, Hilden, Germany), TaqMan™ RNA-to-CT™ 1-Step Kit (catalog # 4392938), STAT1 (catalog # HS 01013996_m1), IFIT3 (catalog #HS01922752752_s1), GAPDH (catalog # Hs02786624_g1), and TaqMan™ PCR Master mix (catalog # 4304437, Thermo Fisher). A CellTiter 96^®^ Aqueous One Solution Cell Proliferation Assay (Catalog # G3582; Promega, WI, USA), a BioTek Synergy HT multimode microplate reader (BioTek, VT, USA) and a Pur-A-Lyzer™ Maxi 6000 Dialysis Kit (Sigma–Aldrich) were purchased from the listed vendors. Poly-D-lysine-coated coverslips (catalog # NC0672873, Fisher Scientific), 4% paraformaldehyde (catalog # J61899. AK), Fluoromount-GTM DAPI (Catalog# 010001, SouthernBiotech), RIPA Lysis and Extraction Buffer (Catalog # 89901), Halt protease and phosphatase inhibitor (Catalog # 78440), Pierce™ BCA Protein Assay Kit (Catalog # 23227), prestained protein ladder (Catalog # 26616) from Thermo Scientific), 4–20% Mini-PROTEAN^®^ TGX (Catalog # 4561095, Bio-Rad), Trans-Blot Turbo RTA Mini 0.2 µm PVDF Transfer Kit (Catalog # 1704272, Bio-Rad, California, USA), beta-actin antibody (Catalog # sc-47778), IFIT3 antibody (Catalog # sc-393512), and m-IgGκ BP-HRP (Catalog # sc-516102) from Santa Cruz Biotech, Dallas, Texas, U.S.A. The STAT1 antibody (catalog # 9167s), anti-rabbit IgG, and HRP-linked antibody (catalog #7074s) were purchased from Cell Signaling Technology (Danvers, MA, USA). West Pico PLUS Chemiluminescent Substrate # 34579, Thermo Scientific).

### Cell culture and differentiation of SH-SY5Y cells

Human SH-SY5Y neuroblastoma cells were cultured in a 75 cm^2^ culture flask and the cells were passaged with 0.05% trypsin and 0.02% EDTA solution. For differentiation, SH-SY5Y cells were seeded at a density of 1x10^5^ cells/cm^2^ in a T75 flask in DMEM/F12 containing 5% FBS and 1% penicillin–streptomycin. After 24 hours, the culture medium was replaced with a fresh medium containing 10 μM RA. RA was added every 3 days for a total of 7 days to induce differentiation toward a more neuron-like phenotype (51–53). The cells were kept in a humidified incubator at 37°C and 5% CO_2_ until they reached 80% confluency. Differentiated SH-SY5Y cells at passage 7 or lower were used for further experiments.

### Treatment of SH-SY5Y cells with Tat protein and cART drugs

Three different concentrations of Tat protein (10, 50, and 100 ng/mL) were prepared in EMEM (54–56). SH-SY5Y cells were treated with Tat protein at the desired concentration according to the experimental conditions, and the incubation period was 24 hours for all the experiments. The concentration of 10 ng/mL Tat protein was selected based on previous studies demonstrating that this level induces molecular and functional changes in neuronal cells without causing significant cytotoxicity (55). A 1 mM stock solution was prepared for all drugs, including FTC, DTG, and TDF, using either PBS or DMSO. A dilution was also performed to obtain the desired concentration for the study. All three drugs, as mentioned above, were used in equal proportions to prepare cART.

### Cytotoxicity assay

An MTS (3-(4-dimethylthiazol-2-yl)-5-(3-carboxymethoxyphenyl)-2-(4-sulfophenyl)-2H-tetrazolium, inner salt) assay was performed to determine the cytotoxicity of the Tat protein in SH-SY5Y cells. SH-SY5Y cells were seeded in a 96-well plate for differentiation until they reached 80% confluency and subsequently treated with various concentrations of Tat protein (10, 50, and 100 ng/ml) for 24 hours. After the incubation period, 20 µL of MTS reagent with 100 µL of media was added to each well, and the plates were incubated for an additional hour. The absorbance of each well was measured at 490 nm via a microplate reader. The percentage of cell viability was calculated via the following formula: % cell viability = absorbance of treated cells/absorbance of control cells × 100 (57).

### Exposure of SH-SY5Y cells to HIV-1

The HIV-1_ADA_ virus (strain NIH-ARP- 416) was obtained from the National Institutes of Health HIV Reagent Program. The virus was thawed and briefly vortexed before use. SH-SY5Y cells were exposed to HIV-1 at a concentration of 100 pg/ml. The virus was added to the cells 24 hours after seeding and incubated for 72 hours to allow for viral entry and initial replication. The control wells were treated with an equivalent volume of virus-free medium.

### HIV infection of the humanized mouse model

Eighteen female Hu-NSG-PBMC mice (strain 745557, 6–7 weeks old) were obtained from Jackson Laboratory and assigned to three groups (n=6 each): a control group, which received a solvent mixture; an HIV group, which received the virus; and a third group, which was infected with HIV for 24 hours, followed by cART drugs exposure (22, 23, 58, 59). In this regard it is worth mentioning that our study was designed based on the hu-PBL mice that were obtained from Jackson Laboratory as mentioned in the manuscript. The reason for developing this model exclusively in female is due to more stable engraftment and immune reconstitution (60). In addition, the H-Y antigen which is lacking in female mice can trigger immune response in the transplanted male mice and cause “Graft-versus-Host reaction”. Thus, this model is largely based on the female mice. Experimentally, the mice were acclimatized for 4 days under controlled conditions (12-hour light/dark cycle, 20–22°C, 50–60% humidity) at The University of Texas Rio Grande Valley facility upon arrival. Baseline weights were recorded two days before experimentation (Day -2). On day 1, the HIV and HIV+cART groups were injected intraperitoneally with 200 pg of HIV. On day 0, the HIV+cART group received a cART drugs that was a combination of TDF (182 mg/kg), DTG (11.6 mg/kg) and FTC (9.3 mg/kg), reflecting human equivalent doses (22, 59). Blood samples were taken on days 1, 5, and 14. At the end of the study (day 14), the mice were euthanized with 30-40% Carbon dioxide displacement per minute in the animal’s home cage and heart perfusion to clear the organ blood, followed by immediate organ harvesting, flash freezing, and storage at -80°C in 24-well plates wrapped in paraffin. All experimental procedures were approved by the Institutional Animal Care and Use Committee (IACUC) of The University of Texas Rio Grande Valley. Standard laboratory conditions and *ad libitum* access to diet and water were maintained throughout the study.

### Analysis of HIV-1 infection in humanized mice

Following HIV infection in humanized mice, blood samples were collected on days 1, 5, and 14 to assess the viral load. Viral p24 antigen levels were quantified via an ELISA (ZeptoMetrix, Catalog No. 0801111). To confirm the results of the ELISA, quantitative PCR was performed to target the HIV-1 3-LTR region. The PCR utilized the following primers and probes: forward primer (F-Primer) GCCTCAATAAAGCTTGCCTTGA, reverse primer (R-Primer) GGCGCCACTAGAGATTTT, and a fluorescence-labeled probe (59FAM/AAGTAGTGTGCCCGTCTGTTGTGTGACT/3IABkFQ) (61). This combination allows for specific amplification and detection of HIV-1 genetic sequences, which facilitates the accurate quantification of viral RNA within the samples.

### RNA extraction and RT–qPCR

Total RNA was extracted from the SH-SY5Y cell line via the RNeasy Mini Kit (Qiagen, Hilden, Germany) according to the manufacturer’s instructions. The purity and yield of RNA were evaluated via a DeNovix DS-11 fluorometer, and cDNA was amplified with the TaqMan™ RNA-to-CT™ 1-Step Kit gene-specific primers for human IFIT3 (Assay id Hs01922752_s1, Human STAT1 (Hs01013996_m1), IL-6 (Hs00174131_m1), TNF alpha Hs00174128_m1), IL-10 (Hs00961622_m1), IL1B (Hs01555410_m1), and GAPDH (Hs99999905_m1). Mouse IFIT3 (Mm01704846_s1) and mouse GAPDH (Mm99999915_g1) obtained from Thermo Fisher. The PCR assays were then performed on an RT–PCR system (Applied Biosystems) under the following conditions: 95°C for 10 minutes, 40 cycles of 95°C for 10 seconds, and 60°C for 1 minute. The reaction specificity was confirmed by running appropriate negative controls. The fold change was calculated via normalization of the expression to that of GAPDH via the ΔΔCt method.

### Immunoblotting

Differentiated SH-SY5Y cells cultured in glucose-supplemented DMEM were seeded in 6-well tissue culture plates at a density of 7.5×10^5^ cells/well; cell lysates were prepared in ice-cold RIPA buffer supplemented with 100X Halt protease and phosphatase inhibitor cocktail for 15 min and then centrifuged for 10 min at 14,000 × g and 4°C. The supernatant was removed, and protein concentrations were determined via a BCA protein assay according to the manufacturer’s protocol. A total of 15 µg of protein was loaded into each well. The samples were prepared by diluting the lysates in 4X laminar loading buffer containing 1% sodium dodecyl sulfate with β-mercaptoethanol and heating for 5 min at 90°C. The samples were processed on 4–12% TGX protein gels (Bio-Rad) at 100 V for 1 h. The proteins were blotted onto PVDF membranes via the Trans-Blot^®^ Turbo™ Transfer System. The membranes were blocked for 1 h at room temperature in buffer consisting of TBST (0.1% Tween-20, 10 mM Tris-HCl, 150 mM NaCl) and 5% BSA. The membranes were probed with the following primary antibodies: mouse anti-IFIT3 (1:500) and the beta-actin antibody phospho-Stat1 (Tyr701) overnight at 4°C. The membranes were washed 3 times for 5 minutes at room temperature with TBS-T and then incubated with horseradish-conjugated anti-mouse or anti-rabbit secondary antibodies. The membrane was developed using West Pico PLUS Chemiluminescent Substrate. Images were captured using an Alliance Q9 Advanced.

### Immunohistochemistry

Immunohistochemical analysis of humanized mouse brain tissue sections was performed according to the protocol (62). In summary, for fixation, mouse tissue was fixed in 10% neutral buffered formalin (NBF) (EprediaTM HiPurTM, Catalog No. 22-110-873) for 48 hours. Embedding: Fixed samples were immersed in melted paraffin (paraplast Plus, melting point 60 to 65°C, Fisher Scientific) three times (1 hour each) and embedded in paraffin with microcassettes and molds. Paraffin-embedded tissue samples were sectioned at a thickness of 4 μm on a rotary microtome and affixed to glass slides (SuperfrostTM Plus, Thermo Fisher, Waltham, MA, USA) in a 40°C hot water bath. Deparaffinization and rehydration: Tissue sections were deparaffinized with xylene and rehydrated through a progressive series of ethanol (50%, 75%, 95%, and 100%). Washing: The sections were washed three times (10 min each) in phosphate-buffered saline (PBS, Fisher Chemicals, Hampton, NH). For blocking, 1% bovine serum albumin (BSA; Fisher Scientific) was added to the samples, which were then incubated with PBS at RT for 1 hour to block nonspecific interactions. For primary antibody incubation, the slides were incubated with a mouse anti-IFIT3 primary antibody (Cat no: 15201-1-AP, Proteintech, Rosemont, IL, USA) at a 1:100 dilution and an anti-NeuN antibody, clone A60 (MAB377, MilliporeSigma, Burlington, MA, USA), at 4°C overnight. For secondary antibody incubation, after primary antibody incubation, the slides were allowed to reach RT for 15 minutes and then washed with PBS. The tissue sections were then incubated with anti-rabbit IgG (H+L) (catalog # A-11008) conjugated with Alexa Fluor 488 or Alexa Fluor 594 at a dilution of 1:500 at RT for 2 hours. After secondary antibody incubation, the slides were washed three times (10 min each) in PBS, and additional washes with PBS were performed to remove any unbound secondary antibodies. Nuclear staining and mounting of the cell nuclei were performed with Fluoromount-GTM DAPI (catalog# 010001, Southern Biotech), and the coverslips were mounted on microscope slides. Image acquisition and analysis: Image acquisition was performed via a Fluoview fV10i fluorescence confocal microscope with 60X Zoom for image capture. The analysis and quantification of the stain intensity, as well as the colocalization studies, were performed via ImageJ (NIH) software.

### Immunocytochemistry

The following steps were performed to prepare the slides for immunofluorescence staining. Cells were seeded on poly-L-coated glass coverslips in 24-well plates and allowed to differentiate with RA, as previously described. The cells were treated with Tat and cART or with cART alone for 24 hours, then fixed with 4% paraformaldehyde in PBS for 10 minutes at RT and washed three times with PBS. The cells were permeabilized, blocked, and washed with 0.1% Triton X-100 in PBS for 10 min and incubated in blocking buffer at RT for 30 min. Following blocking, the cells were washed four times with 0.1% Triton X-100 in PBS for 5 min. The cells were incubated in 0.1% Triton X-100 in PBS containing primary antibodies (1:200 to 1:500 dilution) overnight at 4°C. The slides were subsequently washed three times with 0.1% Triton X-100 in PBS. For secondary antibody incubation, secondary antibodies conjugated with Alexa Fluor 488 were added at a 1:500 dilution to cover glasses and incubated for 1 hour in the dark at RT. For counterstaining and mounting, the cell nuclei were counterstained with DAPI. Images were acquired with an Olympus fluoview fv10i confocal microscope using a 60 × objective. Image analysis was performed with ImageJ (NIH) software to analyze protein expression.

### Machine learning of immunocytochemistry and immunohistochemistry

Convolutional neural networks (CNNs) are well known for productive image classification in various fields and it also provided the best accuracy of the model (63). In addition, we have also employed rigorous validation strategies to ensure reliability of this method, and they are cross-validation, data augmentation and generalization assessment. Experimentally, we utilized such CNN models to perform image classification for the Tat experiments. An architecture of 4 blocks is used, where the typical elements in order are the convolutional layer of filter size 3, batch normalization, the rectified linear unit (ReLU) layer, and an average pooling layer of pool size 2. The convolutional layer channels were 8, 16, 32 and 32. For the last two blocks, the average pooling layer is removed. For the last block, additional layers were added after the ReLU layer, which are the 20% dropout layer, a fully connected layer with several neurons matching the size, a Softmax layer, and a classification layer. This type of architecture has been tested in other works, is practical for many classification tasks and is robust to noise (64). In this study, we employed two primary data augmentation methodologies. The first data augmentation methodology involves rotating images at specific angles (30, 15, 10, and 5 degrees) to increase the dataset size by 18 times the original number. The dataset was then partitioned, with 80% allocated for training and 20% allocated for validation. The validation set sizes were varied (20, 40, 60, and 120), corresponding to each angle partition. Initial experiments were conducted using a single angle for data augmentation, which was adjusted in subsequent trials to improve memory efficiency. All the experiments were performed with 100 epochs and a learning rate of 0.0001, except for the last attempt, which utilized 500 epochs and a learning rate of 0.00001. The second data augmentation methodology consists of parameters like those used in the first methodology for preliminary augmentation. A notable difference was the introduction of a secondary augmentation performed at each epoch during training, where MATLAB’s data augmenter was used to rotate training set images randomly. This approach aims to achieve high augmentation levels without significant memory resource sacrifices. The learning rate for the second methodology was fixed at 0.0001 since it performed better and converged adequately. The second methodology was chosen with the preliminary 15-degree rotation since it demonstrated a nuanced impact on model accuracy and validation performance, with higher angles having varied levels of effectiveness and finer angles increasing the computational time. The learning rate and the number of epochs yielded notable differences in model outcomes. Hyperparameter validation was carried out, and it was found that a learning rate of 0.0001 and setting the number of epochs to 100 models converged with better performance. Importantly, after data augmentation, a partition of 80% for training and 20% for testing was carried out. Owing to the dataset being four times larger than in the previous experiment, images were reshaped to a resolution of 256x256 to address computational constraints. This adjustment significantly reduced the processing time from approximately 10 hours to 45 minutes per run without compromising the accuracy of the predictions, which achieved approximately 100% accuracy across all classes.

### Measurement of reactive oxidative species

SH-SY5Y cells were differentiated in 96-well plates with 10 μM RA. SH-SY5Y cells were incubated for 2 hours with 25 μM H2DCF-DA. After incubation, the H2DCF-DA solution was removed, and the cells were treated with TDF (1.8 μM), DTG (20 μM), FTC (21 Mm) H_2_O_2_, or catalase in their respective wells. The plate was immediately placed in a Bio Tek plate reader (Biotek, Synergy HTX) to measure ROS production in RFU via 480/20 excitation emission of 528/20 (65).

### Statistical analysis

Statistical analyses were carried out with multiple replicates, and the data presented as means ± standard errors of the means. Before the primary statistical analysis, the normality of the data distribution was assessed. Upon confirmation of normality, one-way ANOVA and t tests were used to determine whether there were statistically significant differences between the means of the study groups. To determine the statistical significance of each experiment, one-way analysis of variance (ANOVA) and Dunnett’s multiple comparison tests were conducted via GraphPad Prism version 9 (GraphPad, Inc., CA, USA). A p value of <0.05 was considered to indicate statistical significance.

## Data Availability

The original contributions presented in the study are included in the article/supplementary material. Further inquiries can be directed to the corresponding author.
